# Multi-omics analysis of naïve B cells of patients harboring the C104R mutation in TACI

**DOI:** 10.3389/fimmu.2022.938240

**Published:** 2022-08-16

**Authors:** Neftali Ramirez, Sara Posadas-Cantera, Niko Langer, Andres Caballero Garcia de Oteyza, Michele Proietti, Baerbel Keller, Fangwen Zhao, Victoria Gernedl, Matteo Pecoraro, Hermann Eibel, Klaus Warnatz, Esteban Ballestar, Roger Geiger, Claudia Bossen, Bodo Grimbacher

**Affiliations:** ^1^ Institute for Immunodeficiency, Center for Chronic Immunodeficiencies, Medical Center – University Hospital Freiburg, Faculty of Medicine, Albert-Ludwigs-University, Freiburg, Germany; ^2^ Center for Chronic Immunodeficiency, University Medical Center Freiburg, Freiburg, Germany; ^3^ Department of Rheumatology and Clinical Immunology, Hannover Medical University, Hannover, Germany; ^4^ Resolving Infection Susceptibility (RESIST) – Cluster of Excellence 2155, Hanover Medical School, Satellite Center Freiburg, Freiburg, Germany; ^5^ Department of Rheumatology and Clinical Immunology, Faculty of Medicine, Medical Center - University of Freiburg, Freiburg, Germany; ^6^ Medical Epigenomics & Genome Technology, Research Center for Molecular Medicine(CeMM) of the Austrian Academy of Sciences, Vienna, Austria; ^7^ Institute for Research in Biomedicine, Università della Svizzera italiana, Bellinzona, Switzerland; ^8^ Epigenetics and Immune Disease Group, Josep Carreras Leukaemia Research Institute (IJC), Badalona, Spain; ^9^ Institute of Oncology Research, Università della Svizzera italiana, Bellinzona, Switzerland; ^10^ Deutsches Zentrum für Infektionsforschung (DZIF) – German Center for Infection Research, Satellite Center Freiburg, Freiburg, Germany; ^11^ Centre for Integrative Biological Signalling Studies (CIBSS), Albert-Ludwigs University, Freiburg, Germany

**Keywords:** TACI, CVID, RNA-seq, ATAC-seq, proteomics, transcription factors, NF-kB

## Abstract

Common variable immunodeficiency (CVID) is the most prevalent form of symptomatic primary immunodeficiency in humans. The genetic cause of CVID is still unknown in about 70% of cases. Ten percent of CVID patients carry heterozygous mutations in the tumor necrosis factor receptor superfamily member 13B gene (*TNFRSF13B*), encoding TACI. Mutations in *TNFRSF13B* alone may not be sufficient for the development of CVID, as 1% of the healthy population carry these mutations. The common hypothesis is that TACI mutations are not fully penetrant and additional factors contribute to the development of CVID. To determine these additional factors, we investigated the perturbations of transcription factor (TF) binding and the transcriptome profiles in unstimulated and CD40L/IL21-stimulated naïve B cells from CVID patients harboring the C104R mutation in *TNFRSF13B* and compared them to their healthy relatives with the same mutation. In addition, the proteome of stimulated naïve B cells was investigated. For functional validation, intracellular protein concentrations were measured by flow cytometry. Our analysis revealed 8% less accessible chromatin in unstimulated naïve B cells and 25% less accessible chromatin in class-switched memory B cells from affected and unaffected TACI mutation carriers compared to healthy donors. The most enriched TF binding motifs in TACI mutation carriers involved members from the ETS, IRF, and NF-κB TF families. Validation experiments supported dysregulation of the NF-κB and MAPK pathways. In steady state, naïve B cells had increased cell death pathways and reduced cell metabolism pathways, while after stimulation, enhanced immune responses and decreased cell survival were detected. Using a multi-omics approach, our findings provide valuable insights into the impaired biology of naïve B cells from TACI mutation carriers.

## Graphical Abstract

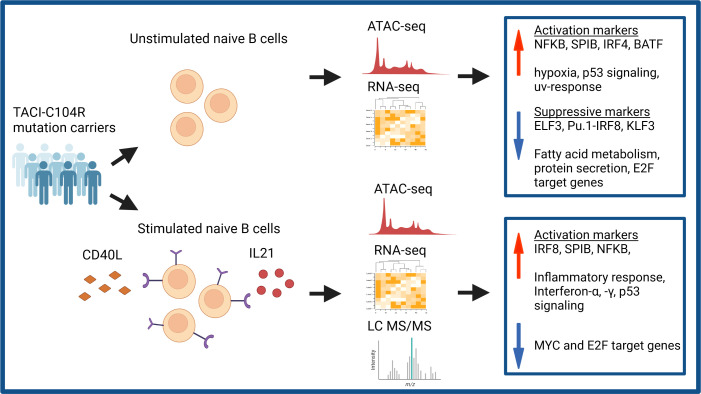

## Introduction

Common variable immunodeficiency (CVID), the most prevalent primary immunodeficiency in humans with an incidence of 1 in 50,000 to 1 in 10,000 Caucasians, forms a heterogeneous group of disorders (1). CVID is characterized by recurrent bacterial infections, hypogammaglobulinemia, and impaired antibody responses pointing to a defect in B cell differentiation or activation (2). To date, the cause of CVID and its development has not been identified in all patients, as only ~25% have monogenetic alterations explaining their phenotype (3, 4). Approximately 10% of CVID patients harbor a mutation in *TNFRSF13B* (encoding the protein TACI) with a demonstrable biological impact (5), but 1%–2% of the healthy population also carry these variants, causing debate on whether *TNFRSF13B* mutations should be termed “disease causing” or merely “disease associated”.

Transcriptional activation occurs as a result of binding of transcription factors (TFs) and the acquisition of a competent epigenetic status, which also associates with increased chromatin accessibility (6). ATAC-seq (assay for transposase accessible chromatin and sequencing) determines the accessible chromatin regions of a given cell population (often about 50,000 cells) genome-wide. Using ATAC-seq, Yang et al. identified functional genes not previously highlighted as causative for rheumatoid arthritis (7), demonstrating the benefit of chromatin investigations.

Transmembrane activator and calcium modulator and cyclophilin ligand interactor (TACI, CD267) is encoded by *TNFRSF13B* and is a type III transmembrane protein belonging to the TNFR family. TACI plays a role in multiple events in the immune response by regulating the B cell pool size through a negative feedback loop; it induces IgG and IgA class switch recombination in germinal center (GC) B cells and promotes plasma cell survival and differentiation (8, 9). Upon ligand binding of the B cell activating factor (BAFF) or a proliferation-inducing ligand (APRIL), the intracellular domains of TACI interact with multiple signaling and activation factors, inducing activation of the canonical (classical) nuclear factor kappa-B (NF-κB) signaling pathway (10). Thus, TACI is a crucial receptor for B cell maturation and is upregulated in activated B cells. Class-switch memory B cells and plasma cells express high levels of TACI, while transitional, naïve, and marginal zone B cells show lower expression (11).

The most common TACI mutations in CVID are C104R and A181E, which affect ligand binding and transmembrane function, respectively (12–14). Homozygous C104R carriers have fewer autoimmune manifestations compared to heterozygous C104R CVID patients, and a milder phenotype (5). In CVID patients harboring a single C104R TACI mutation, a diminished immune tolerance and a high titer of IgG antinuclear antibodies (ANAs) associated with activated circulating T follicular helper cells, possibly driving autoreactive B cells, were observed (15). In addition, Crotty et al. demonstrated a decreased TLR9 response to CpG in B cells of CVID patients carrying the C104R mutation in TACI (16). Furthermore, in naïve B cells from CVID patients with and without TACI mutations, elevated proliferation was observed compared to healthy donors with and without TACI mutations (16). Diminished IgA titers are associated with TACI mutations (17) and observed in 13 from our 18 C104R mutation carriers (**Table 1**).

**Table 1:** Clinical overview of all investigated individuals.

Index patient	Sex	Age	Zygosity	Condition	IgG in g/L	IgA in g/L	IgM in g/L	Autoimmune complications	Ig substitution	Infections	Other complications
				normal reference value (95% CI)	7-16	0,7-4	0,4-2,3				
TACI_1.1	male	70	heterozygous	unstimulated	n.a.	n.a.	n.a.	n.a.	n.a.	n.a.	n.a.
TACI_1.2	female	38	heterozygous	unstimulated	n.a.	n.a.	n.a.	n.a.	n.a.	n.a.	n.a.
TACI_2.1	female	24	heterozygous	unstimulated	13.6	1.24	0.57	no	yes	recurrent respiratory infections	no
TACI_2.2	female	57	heterozygous	unstimulated	n.a.	n.a.	n.a.	rheumatic complaints	no	recurrent respiratory infections	no
TACI_3.1	female	77	heterozygous	unstimulated	9.8	<0.1	<0,1	no	yes	no	no
TACI_3.2	female	81	heterozygous	unstimulated	7.9	1.1	0.7	no	no	sinus chest infections	no
TACI_4	male	34	homozygous	unstimulated	5.29	0.28	<0,17	no	yes	bronchitis	splenomegaly
TACI_5	female	58	homozygous	unstimulated	n.a.	n.a.	n.a.	n.a.	n.a.	n.a.	n.a.
TACI_6	male	20	homozygous	unstimulated	n.a.	n.a.	n.a.	n.a.	n.a.	n.a.	n.a.
TACI_7.1	male	56	heterozygous	unstimulated	7.51	0.41	0.43	Sicca-syndrom	yes	recurrent respiratory infections	splenomegaly, chronic diarrhea
TACI_7.2	male	84	heterozygous	unstimulated	n.a.	n.a.	n.a.	n.a.	n.a.	n.a.	n.a.
TACI_8.1	male	38	heterozygous	unstimulated	10.6	1.46	0.45	enteropathy	yes	recurrent gastrointestinal infections	chronic diarrhea
TACI_8.2	female	66	heterozygous	unstimulated	8	0.7	0.52	no	yes	no	no
TACI_9.1	female	45	heterozygous	unstimulated	10.3	<0,25	<0,17	urtikaria	yes	recurrent pneumonia	no
TACI_9.2	female	79	heterozygous	unstimulated	n.a.	n.a.	n.a.	n.a.	n.a.	n.a.	n.a.
TACI_10	male	31	heterozygous	stimulated	13.8	< 0,06	< 0,17	thyroiditis	yes	recurrent sinusitis, bronchitiden, pharyngitis	no
TACI_11	female	60	heterozygous	stimulated	12.1	< 0,06	< 0,17	enteropathy	yes	recurrent sinusitis, bronchitiden, multiple pneumonias	splenomegaly, lymphadenopathy, chronic diarrhea
TACI_12	male	65	heterozygous	stimulated	12.9	0.98	0.43	asthma	yes	sinusitis, bronchitis	no
TACI_13	female	65	heterozygous	stimulated	11	0.26	0.43	no	yes	recurrent respiratory infections	breast cancer
TACI_7.1	male	55	heterozygous	stimulated	9.13	0.48	0.49	Sicca-syndrom	yes	recurrent respiratory infections	splenomegaly, chronic diarrhea
TACI_14	male	53	heterozygous	stimulated	1.44	< 0,06	< 0,17	vitiligo	no	recurrent respiratory infections, chronic sinusitiden	splenomegaly
TACI_15	female	31	heterozygous	stimulated	11	0.24	0.81	no	yes	sinusitis, bronchitis, otitis	no
TACI_16	male	40	heterozygous	stimulated	13	0.09	0.42	enteropathy	yes	sinusitis, bronchitis, parotitis	splenomegaly, ILD, lymphadenopathy
TACI_17	female	44	heterozygous	stimulated	6.1	0.5	2.92	urtikaria	no	sinusitis, bronchitis	ILD, morbus bowen
TACI_18	male	35	heterozygous	stimulated	7.33	< 0,06	1.6	cytopenias, enteropathy	yes	recurrent respiratory infections	splenomegaly, myelitis
HD1	female	59	wild-type	unstimulated	n.a.	n.a.	n.a.	no	no	no	no
HD2	male	63	wild-type	unstimulated	n.a.	n.a.	n.a.	no	no	no	no
HD3	female	25	wild-type	unstimulated	n.a.	n.a.	n.a.	no	no	no	no
HD4	male	41	wild-type	unstimulated	n.a.	n.a.	n.a.	no	no	no	no
HD5	female	27	wild-type	unstimulated	n.a.	n.a.	n.a.	no	no	no	no
HD6	male	51	wild-type	unstimulated	n.a.	n.a.	n.a.	no	no	no	no
HD7	male	51	wild-type	unstimulated	n.a.	n.a.	n.a.	no	no	no	no
HD8	male	52	wild-type	unstimulated	n.a.	n.a.	n.a.	no	no	no	no
HD6.2	male	52	wild-type	stimulated	n.a.	n.a.	n.a.	no	no	no	no
HD9	male	29	wild-type	stimulated	n.a.	n.a.	n.a.	no	no	no	no
HD10	female	35	wild-type	stimulated	n.a.	n.a.	n.a.	no	no	no	no
HD11	female	30	wild-type	stimulated	n.a.	n.a.	n.a.	no	no	no	no
HD12	female	53	wild-type	stimulated	n.a.	n.a.	n.a.	no	no	no	no
HD13	female	39	wild-type	stimulated	n.a.	n.a.	n.a.	no	no	no	no
HD14	male	25	wild-type	stimulated	n.a.	n.a.	n.a.	no	no	no	no
HD15	male	18	wild-type	stimulated	n.a.	n.a.	n.a.	no	no	no	no
HD16	female	25	wild-type	stimulated	n.a.	n.a.	n.a.	no	no	no	no
HD17	male	32	wild-type	stimulated	n.a.	n.a.	n.a.	no	no	no	no
HD18	female	40	wild-type	stimulated	n.a.	n.a.	n.a.	no	no	no	no
HD4.2	male	43	wild-type	stimulated	n.a.	n.a.	n.a.	no	no	no	no
HD19	female	28	wild-type	stimulated	n.a.	n.a.	n.a.	no	no	no	no
HD20	female	33	wild-type	stimulated	n.a.	n.a.	n.a.	no	no	no	no
HD21	male	33	wild-type	stimulated	n.a.	n.a.	n.a.	no	no	no	no
HD22	female	47	wild-type	stimulated	n.a.	n.a.	n.a.	no	no	no	no
HD2.2	male	65	wild-type	stimulated	n.a.	n.a.	n.a.	no	no	no	no
HD23	female	26	wild-type	stimulated	n.a.	n.a.	n.a.	no	no	no	no

n.a., information not available, either data was not collected or follow up was lost.

ILD, interstitial lung disease.

Multiple studies observed progressive disease development in CVID patients harboring the C104R mutation. Patients suffer from an increased risk for autoimmune manifestations, independent of zygosity (hetero- or homozygous mutation carrier) or disease penetrance and expressivity (CVID or healthy individuals) (5, 15). In addition, malignancies, such as lymphoma and gastric cancer (18), are associated with TACI mutations, as well as splenomegaly (19). A common complication reported for TACI-CVID patients is interstitial lung disease, a major cause for mortality (20). In addition to these reported complications in TACI CVID patients, healthy C104R mutation carriers seem to be prone to autoimmunity (15), highlighting the complex involvement of TACI in immune homeostasis.

The common hypothesis is that TACI mutations are not fully penetrant and additional factors contribute to the development of CVID. To determine these additional factors, we investigated the perturbations of TF binding and the transcriptome profiles in CVID patients harboring the C104R TACI mutation and compared them to their healthy relatives harboring the same mutation and to healthy donors tested negative for the mutation. In addition, we analyzed the chromatin landscape by ATAC-Seq, the transcriptome by bulk-RNA-Seq, and proteome by mass spectroscopy of sorted and stimulated naïve B cells from CVID patients.

## Materials and methods

### Study participants

In this study, 19 individuals (16 heterozygous and 3 homozygous *TNFRSF13B* c.310 T>C mutation carriers; further referred to as affected *TNFRSF13B* mutation carriers) previously diagnosed with CVID, 6 healthy relatives of 6 CVID patients harboring the same heterozygous *TNFRSF13B* mutation (further referred to as unaffected *TNFRSF13B* mutation carriers), and 26 healthy donors tested *TNFRSF13B* wild type (wt) were recruited. One heterozygous patient and relative are residents of the United Kingdom, another in Spain, a third in the Netherlands, and the remaining heterozygous patients and their relatives are residents of Germany. Two homozygous patients are residents of Spain and one in Germany. The residency of all healthy donors was Germany. All investigations were performed after individuals have signed the informed consent form from their local physician [V2, 12/19, D; MEC-2013-026, NL; PR(AG)12/2016, S; and 04/Q0501/119, UK]. The ethics committee of Freiburg approved this project with an effective date of 6 June 2019. In July 2019, the consent form under which patient samples had been collected was approved under ETK-Nr_354/19. The i-PAD ethics vote carries the number EK-Freiburg_76/19.

### Cell isolation and stimulation

Whole blood or frozen peripheral blood mononuclear cells (PBMCs) (36–72 ml) were collected from all individuals either from Freiburg University Clinic or shipment from collaborators. PBMCs were isolated using standard Ficoll density centrifugation in 50 ml Falcon or Sepmate tubes (Stemcell Technologies). PBMCs were stained with trypan blue or Nigrosin and counted under the microscope with a “Neubauer Chamber”. A total of 100,000 PBMCs per condition (B cell panel and B cell activation panel) were stained and measured by flow-cytometry (see *FACS staining*). For unstimulated experiments, B cells were separated from the remaining PBMCs using microbeads (MACS^®^ CD19^+^ microbeads, Miltenyi Biotec). Cells were centrifugated for 10 min, at 300 g and 8°C, and the cell pellet was resuspended in 80 µl of MACS buffer (PBS + 0.5% BSA and 2 mM EDTA) and 20 µl of CD19 microbeads. The cell suspension was incubated for 15 min at 4°C and washed with 10 ml of MACS buffer for 10 min, at 300 g and 8°C, before they were applied to the MACS column. The flow through containing the non-B cells was collected in RMPI medium + 10% FCS and 1% penicillin/streptomycin, frozen in 90% RPMI with adjuvants + 40% FCS + 10% DMSO, and stored in the N2 tank. CD19^+^ B cells were flushed out of the column with 1 ml of FACS buffer and stained with B cell panel to sort naïve (CD19^+^CD20^+^CD21^+^CD27^-^CD38^+^IgM^+^IgD^+^) and class-switched memory B cells (CD19^+^CD20^+^CD21^+^CD27^+^IgM^-^IgD^-^) on a MoFlo Astrios (Beckman Coulter). For stimulation experiments, naïve B cells (CD14^-^CD4^-^CD19^+^CD27^-^IgD^+^) were sorted directly from PBMCs using the sort panel. B cells (0.25 Mio/ml) were stimulated in RPMI in a 96-well plate (round bottom) for 24 h at 37°C, 5% CO_2_ with 1 µg/ml recombinant CD40L (provided by Pascal Schneider, Lausanne, Switzerland) + 50 ng/ml IL21 (Miltenyi).

### FACS staining

For intra- and extracellular stainings, cells were centrifugated for 5 min, at 300 g at RT, and the supernatant was discarded. The cell pellet was resuspended with 50 µl (FACS panel) or 100 µl (for sorting) of the corresponding antibody master mix containing optimal dilutions of antibodies, for 30 min at 4°C. After washing the cells, the cell pellet was resuspended in 300 µl of FACS buffer with propidium iodide (BioLegend), a stain for viable cells and analyzed using an LSR Fortessa (BD Biosciences).

Sort panel: IgD PE (IA6-2), CD4 APC-Cy7 (RPA-T4), CD19 Brilliant Violet 510 (HIB19), and CD27 Brilliant Violet 421 (M-T271), all from BioLegend and CD14 APC (M5E2) from BD Biosciences. The panel was used to exclude T cells and monocytes, and collect IgD+ naïve B cells, adopted from Warnatz et al. (21); stimulate them with CD40L and IL21 for 24 h.

B cell panel: CD19 APC-Cy7 (HIB19), CD20 APC (2H7), CD21 Pe-Cy7 (BU32), CD38 PerCP-Cy5.5 (HIT2), CD27 Brilliant Violet 421 (M-T271), IgD PE (IA6-2), and IgM Alexa Fluor 647 (MHM-88), all from BioLegend. The panel was used to sort naïve- and class-switched memory B cells (22) for ATAC- and RNA-seq.

B cell activation panel: CD80 Brilliant violet 421 (2D10), CD86 PerCP-Cy5.5 (IT2.2), CD95 Brilliant violet 650 (DX2), and HLA-DR PE-Cy7 (L243), all from BioLegend, and CD69 APC (L78) from BD Biosciences. The panel was used to assess the success of B-cell stimulation (23).

IκBα panel: CD19 Brilliant Violet 421 (HIB19), CD21 Pe-Cy7 (BU32), and CD38 PerCP-Cy5.5 (HIT2), all from BioLegend; CD27 Brilliant Violet 605 (L128) and IκBα PE (MAD-3) were from BD Biosciences; IgD FITC (F(ab’)2) was from Southern Biotech; and IgM Alexa Fluor 647 (F(ab’)2) was from Jackson Immunoresearch laboratories. The panel was used to determine IκBα expression in naïve B cells (24).

pERK panel: CD19 Brilliant Violet 421 (HIB19), CD21 Pe-Cy7 (BU32), and CD38 PerCP-Cy5.5 (HIT2), all from BioLegend; CD27 Brilliant Violet 605 (L128) and pERK Alexa Fluor 647 were from BD Biosciences; and IgD FITC (F(ab’)2) was from Southern Biotech. The panel was used to determine pERK expression in naïve B cells. The panel was adopted from Keller et al. (24).

### IκBα degradation assay

IκBα degradation was determined by intracellular FACS staining, as described before (24). Fresh PBMCs (5 × 0.5 million) per individual were incubated in 0.5 ml of RPMI overnight at 37°C and 5% CO_2_. The next day, the cells were stimulated with four different stimuli. Zombie UV (1 µl; BioLegend) was added to each tube, vortexed, and incubated for 15 min at RT, 5% CO_2_. Then, for the stimulations, 50 µl of RPMI containing the double-concentrated stimulus was added to the FACS tubes. Either 15 mg/ml F(ab)2 goat anti-human IgM (SouthernBiotech, Birmingham, Ala), 5 mg/ml CpG-ODN 2006 (Invivogen, Toulouse, France), 1 µg/ml CD40L (provided by Pascal Schneider, Lausanne, Switzerland), 200 ng/ml phorbol 12-myristate 13-acetate (PMA; Sigma-Aldrich, St Louis, MO), or 50 µl of RPMI was added, as indicated. After stimulation (anti-IgM: 45 min, CpG: 1 h, PMA and CD40L: 15 min), cells were fixed using 100 µl of Cytofix and incubated for 10 min at RT, 5% CO_2_. After centrifugation for 5 min, at 1,200 rpm at RT, the supernatant was discarded and cells were permeabilized with 380 µl of Perm Buffer III (both from BD Biosciences, San Jose, CA) on ice for 30 min. After washing cells twice with PBS + 0.5% BSA, they were stained 30 min at RT in the dark with the appropriate antibodies (see FACS staining, IκBα panel). Cells were washed with PBS + 0.5% BSA, followed by resuspension of the cells in 200 µl of RPMI and then measured on an LSR Fortessa.

### Phosphorylated ERK expression assay

Frozen PBMCs were thawed, washed twice with RPMI medium, and centrifuged for 5 min, at RT and 300 g, followed by cell counting under the microscope, as previously described. PBMCs (5 × 0.5 million) were incubated overnight at 37°C with 5% CO_2_. The same protocol was used for the IκBα degradation assay. PBMCs were stimulated with 1 µg/ml recombinant CD40L, for 8 min or measured unstimulated on an LSR Fortessa.

### ATAC-sequencing

Accessible chromatin mapping for unstimulated cells was performed using the ATAC-seq method as previously described (25), with minor adaptations. Between 850 and 5 × 10^4^ cells were centrifuged for 10 min at 8°C, resuspended in 50 µl of lysis buffer (10 mM Tris-HCl, pH 7.4, 10 mM NaCl, 3 mM MgCl_2_, and 0.1% NP-40) and centrifuged for 30 min at 500 g and 8°C. Next, the pellet was incubated in 25 µl of transposase reaction mix [12.5 µl of 2× TD buffer, 1 µl of transposase (Illumina), and 11.5 µl of nuclease-free water] for 60 min at 37°C. After DNA purification with the Clean and Concentrator kit (Zymoresearch), DNA was eluted in 13.5 µl. Ten microliters of the eluted DNA was used in a 50-µl quantitative PCR reaction, and after five cycles, 5 µl of the eluted DNA was used in a quantitative PCR (qPCR) reaction to estimate the optimum number of amplification cycles. The remaining 45 µl was amplified for the determined cycle number, followed by two rounds of AMPure XP bead (Agencourt) size selection. DNA concentration was measured with the high-sensitivity Qubit fluorometer kit (Life Technologies). The quality was validated using the Agilent Technologies 2200 Tape Station System (High sensitivity DNA kit). The libraries were sequenced by the Genomic Core Sequencing Facility at EMBL using the Illumina HiSeq 2500 platform and the 50-bp single-end configuration. For stimulated cells, 50,000 cells were centrifuged for 5 min at 500 g and 8°C and resuspended in transposase buffer [12.5 µl of 2× TD buffer, 1 µl of transposase (Illumina), 0.25 µl of 1% Digitonin (Promega), 0.5 µl of 50× PIC (Roche), and 10.75 µl of nuclease-free water]. After 30 min of incubation at RT, the DNA clean and concentrator kit was used and samples were stored at −80°C. Further library preparation and sequencing was performed at the Biomedical Sequencing Facility at CeMM in Vienna using the Illumina HiSeq 4000 platform and the 50-bp single-read configuration (26). One microliter of the eluted DNA was used in a quantitative PCR reaction to estimate the optimum number of amplification cycles. Library amplification was followed by SPRI size selection to exclude fragments larger than 1,200 bp. DNA concentration was measured with a Qubit fluorometer (Life Technologies). Library amplification was performed using custom Nextera primers.

### Processing of the ATAC-seq data

Reads from unstimulated cells were aligned to the GRCh38/hg38 assembly of the human genome using Bowtie (27) with the –best and -m 1 parameters, while stimulated cells were aligned to hg38 using Bowite2. All downstream analyses were performed on the filtered reads (26). In total, more than 100 million sequenced fragments were obtained for the libraries generated, and reads mapping to mitochondrial DNA were excluded from the analysis. Peaks were called using HOMER (28) with parameters -style factor and -L 20. Differential peaks were identified using getDifferentialPeaksreplicates.pl -balanced –edgeR -L 10. Peaks were merged and annotated with annotatePeaks.pl. One non-annotated file was generated with the option -noann, uploaded to Rstudio, and, with the package ggplot2 (version 3.3.5), principal component analysis was performed. Motif analysis was performed by HOMER function findMotifsGenome.pl with the parameters -size 200 and -len 8,10,12,15. With the -size 200 -hist 400 -ghist parameters from HOMER, a data matrix file was generated and uploaded to Cluster 3.0 for unsupervised hierarchical clustering and visualized with Java Tree View.

### RNA-sequencing

Between 22,000 and 50,000 cells were centrifuged for 5 min at 8°C and the supernatant was discarded. The cell pellet of unstimulated cells was resuspended in either 350 µl of RA1 buffer (Macherey-Nagel) or 350 µl of RLT buffer (Qiagen) with 3.5 µl of β-mercaptoethanol followed by immediate vortexing for 30 s and storage at −80°C. RNA isolation was performed according to the RNeasy Micro kit protocol (Qiagen) or to the NucleoSpin RNA protocol (Macherey-Nagel). Frozen cells were thawed and vortexed for 30 s. Elution buffer (15–20 µl) from Qiagen was added to the membrane and centrifuged for 1 min at full speed or 11,000 *g*. The eluate was transferred back to the membrane and the centrifugation was repeated. Low DNA library preparation (New England Biolabs) and sequencing was performed by the Genomic Core Sequencing Facility at EMBL using the Illumina NextSeq platform and the 75-bp single-end configuration. The cell pellet of stimulated cells was resuspended in 350 µl of RLT Plus buffer (Qiagen) with 3.5 µl of β-mercaptoethanol followed by immediate vortexing for 30 s and storage at −80°C. RNA isolation was performed according to the AllPrep DNA/RNA Micro kit protocol (Qiagen) and eluted in 15 µl, as described above. RNA library preparation (NEBNext RNA First Strand Synthesis Module; NEBNext Ultra Directional Second Strand Synthesis Module) and sequencing was performed by Novogene (Cambridge, UK) using the Illumina NovaSeq 6000 platform and the 150-bp paired-end configuration.

### Processing of RNA-seq data

The public server from usegalaxy.org was used for the initial analysis (29). First, quality was captured with FastQC (version 0.72+galaxy1) followed by trimming the first 3 bp of each sequence using Trimmomatic. Reads were aligned against the GRCh38/hg38 assembly of the human genome using default single-end (unstimulated cells) or with the modification of the length of the genomic sequence around annotated junctions to 149 and the maximum number of alignments to output a read’s alignment results to 3 for paired-end (stimulated cells) RNA-STAR conditions. Duplicates were not removed. FeatureCounts was used to measure gene expression with unstranded specification and built-in gene annotation. Further analysis was performed with RStudio. First, genes expressed below 100 read counts were excluded. Then, differentially expressed genes were determined using the R Bioconductor package DeSeq2 (version 1.30.1). To account for the batch effect due to sex and isolation kit, the batch was included in the design formula. Next, differentially expressed genes were filtered according to their adjusted *p*-value (<0.05) and absolute value of log2 fold change (>0.5), and principal component analysis was performed. Heatmaps and volcano plots were generated using the R package pheatmap (version 1.0.12) and the Bioconductor package EnhancedVolcano (version 1.8.0), respectively. Gene set enrichment analysis was performed using the R Bioconductor package fgsea (version 1.16.0) (30) together with the Hallmark pathways from the MSigDb. As suggested by fgsea, all differentially expressed genes were used for the analysis. Ggplot2 (version 3.3.5) package was used to create dot plots of pathway analysis.

### Proteome

#### Protein extraction and enzymatic digestion

After stimulation, between 54,000 and 189,000 cells per sample were washed twice in phosphate-buffered saline (PBS), and dry pellets were flash frozen and stored at −80°C. Cell lysis and protein extraction were performed by resuspending each pellet in 50 µl of 8 M urea in 50 mM ammonium bicarbonate (ABC) and sonicating it for 15 min at 4°C (Bioruptor, Diagenode). For each sample, up to 100 µg of protein extract was reduced with 10 mM dithiothreitol for 20 min at room temperature and alkylated with 50 mM iodoacetamide for 30 min at room temperature. Protein digestion with 1 µg of LysC (FUJIFILM Wako) was carried out in 8 M urea and 50 mM ABC for 2 h at room temperature, after which the digestion buffer was diluted to a final 2 M urea with 50 mM ABC, and digestion with 1 µg of trypsin (Promega) was carried out overnight at room temperature. The following day, digestion was stopped by adding acetonitrile (ACN) to 2% and trifluoroacetic acid (TFA) to 0.3% and the samples were cleared by centrifugation for 5 min at maximum speed. The supernatants were then desalted with C18 StageTips, from which purified peptides were eluted with 80% ACN, 0.5% acetic acid. Finally, the elution buffer was evaporated by vacuum centrifugation and purified peptides were resuspended in 2% ACN, 0.5% acetic acid, and 0.1% TFA. For LC-MS/MS analysis, up to 1 µg of purified peptides from each sample was injected as single-shot measurements.

#### LC-MS/MS analysis

Peptides were separated on an EASY-nLC 1200 HPLC system (Thermo Fisher Scientific), coupled online to a Q Exactive HF mass spectrometer (Thermo Fisher Scientific) *via* a nanoelectrospray source (Thermo Fisher Scientific). Peptides were loaded in buffer A (0.1% formic acid) into a column (75 µm inner diameter, 50 cm length) in-house packed with ReproSil-Pur C18-AQ 1.9 µm resin (Dr. Maisch HPLC GmbH), and eluted over a 150-min linear gradient of 5%–30% buffer B (80% ACN, 0.1% formic acid) with a 250 nl/min flow rate. The Q Exactive HF was operated with the Xcalibur software (Thermo Scientific) in a data-dependent mode with a survey scan range of 300–1,650 m/z, a resolution of 60,000 at 200 m/z, a maximum injection time of 20 ms, and an AGC target of 3e6. Up to 10 most abundant ions with charge 2–5 were isolated with a 1.8 m/z isolation window and subjected to higher-energy collisional dissociation (HCD) fragmentation with a normalized collision energy of 27. MS/MS scans were acquired with a resolution of 15,000 at 200 m/z, a maximum injection time of 55 ms, and an AGC target of 1e5. Dynamic exclusion was set to 30 s to avoid repeated sequencing.

#### LC-MS/MS data analysis

Xcalibur raw files were processed with the MaxQuant software, v.1.6.7.0 (31). Searches were performed by the integrated Andromeda search engine (32) against the Human UniProt database (June 2019) and a common contaminants database to identify peptides and proteins with a false discovery rate of <1%. Enzyme specificity was “Trypsin/P” with a maximum of two missed cleavages and a minimum length of seven required for peptide identification. N‐terminal protein acetylation and methionine oxidation were set as variable modifications, while cysteine carbamidomethylation was set as a fixed modification. Match between runs was enabled to transfer identifications across measurements based on mass and normalized retention times, with a matching time window of 0.7 min and an alignment time window of 20 min. Label‐free protein quantification (LFQ) was performed with the MaxLFQ algorithm (33) with a minimum peptide ratio count of 1 required for quantification.

Data were filtered by removing proteins only identified by site, reverse peptides, and potential contaminants, and label-free protein quantification intensities were log2 transformed. Further analysis was performed with RStudio. Normalization and imputation using a left-censored method that replaces missing values taking them from a normal distribution with 0.3 width and 1.8 downshift were applied with the R Bioconductor package DEP (version 1.12.0). Next, differentially expressed proteins were identified using the *t*-test from the R package limma (version 3.46.0) with alpha = 0.05 and an absolute log2 fold change of 0.5. All visualizations were performed as described for the transcriptome analysis.

## Results

### Chromatin landscapes from naïve and class-switched memory B cells from healthy donors and TACI mutation carriers

**Figure 1:**
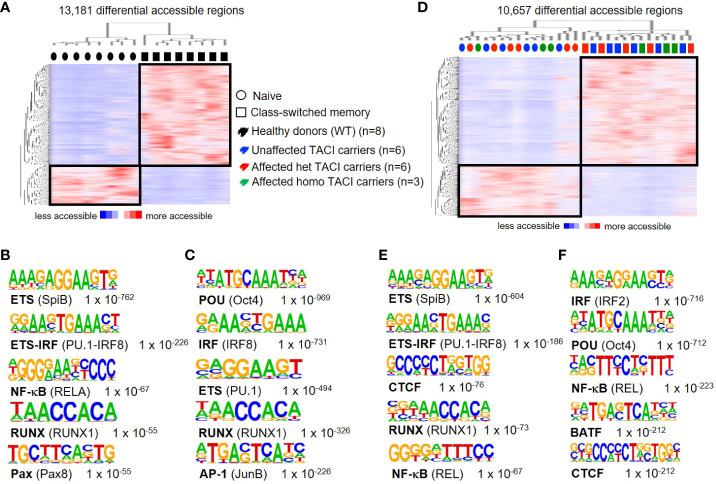
Chromatin accessibility landscapes from unstimulated naïve and CSM B cells from TACI mutation carriers and healthy donors. **(A)** Heatmap from healthy donors including hierarchical clustering of 13,181 differentially accessible chromatin regions in naïve B cells compared to CSM B cells. Preferentially open regions amounted to 3,736 in naïve B cells and 9,445 in CSM B cells. **(B, C)** Cis-regulatory sequences associated with regions of the **(B)** naïve B cells or **(C)** CSM B cells. **(D)** Heatmap from TACI mutation carriers including hierarchical clustering of 10,657 differentially accessible chromatin regions in naïve B cells compared to CSM B cells. Preferentially open regions amounted to 3,467 in naïve B cells and 7,190 in CSM B cells. **(E, F)** Cis-regulatory sequences associated with the regions of the **(E)** naïve B cells or **(F)** CSM B cells. Transcription factor family stated first and in brackets the family member. Genes were normalized and centered with Cluster 3.0 and visualized with Java Treeview with color indicating the region with maximum (red) or minimum (blue) open chromatin. Sequence logos and *p*-values reflecting the significance of motif occurrence are shown next to the corresponding motif.

To assess the accuracy of accessible chromatin regions for B cell activation and differentiation, we first compared accessible chromatin regions of naïve and class-switched memory (CSM) B cells from eight healthy donors, as these B cell subpopulations have different properties. As expected, two distinct clusters formed, distinguishing both B cell subgroups. We detected 13,181 differentially accessible regions (DARs) (identified with 10-fold greater tag density than background), with a third (3,736 regions) preferentially accessible in naïve B cells and two-thirds (9,445 regions) preferentially accessible in CSM B cells (**Figure 1A**). To identify TFs associated with this differential chromatin accessibility, we performed *de novo* motif analysis. Our analysis of motif lengths 8, 10, 12, and 15 bp and peak size 200 identified the ETS motif as the most enriched transcription factor binding motif (TFBM) (**Figure 1B**) in naïve B cells from healthy donors, suggesting that member(s) of the ETS family play an important role in the cis-regulatory network in naïve B cells. PU.1 and SPIB were recently shown to be important for GC formation and class switch recombination and to prevent plasma cell differentiation (34). The second most enriched motif in naïve B cells was the complementary ETS-IRF fusion TF.

In CSM B cells of healthy donors, the first two TFBM were for the TF family POU and IRF, respectively (**Figure 1C**). Oct4 is known to suppress B-cell lineage differentiation and is active in the GC, while IRF8 is an important TF for the generation of GCs and plasma cells (35–37).

A role for these TFs in memory B cells has not been described, and future studies will be necessary to determine the role of these TFBM in memory B cells, as class-switched memory B cells have passed through the GC reaction. Our results are consistent with findings from Moroney et al., who showed that ETS family members are the most prevalent TFBM in naïve B cells, and POU and ETS family member TFBM are most enriched in CSM B cells (38).

To investigate the impairment of B-cell maturation in TACI mutation carriers (six heterozygous and three homozygous affected mutation carriers and six heterozygous unaffected mutation carriers), we analyzed their naïve B-cell and CSM B-cell compartment by ATAC-seq. Similar to our findings in healthy donors, our results revealed 10,657 differentially accessible regions in total, a third (3,467 regions) preferentially open in naïve B cells and two-thirds in CSM B cells (7,190 regions) (**Figure 1D**). Two distinct clusters distinguished naïve B cells from CSM B cells. Interestingly, we found a random distribution of heterozygous and homozygous, affected and unaffected naïve B cells and CSM B cells within the two clusters, indicating a minor influence of the mutational or clinical status. Four of the five most enriched *de novo* motifs detected from naïve B cells were identical to the enriched motifs in the HD comparison (**Figure 1E**), but for the class-switched memory B cell subset, the TF family IRF led the list, followed by the POU family (**Figure 1F**).

### Dysregulated chromatin landscape in unstimulated naïve B cells from TACI mutation carriers

**Figure 2:**
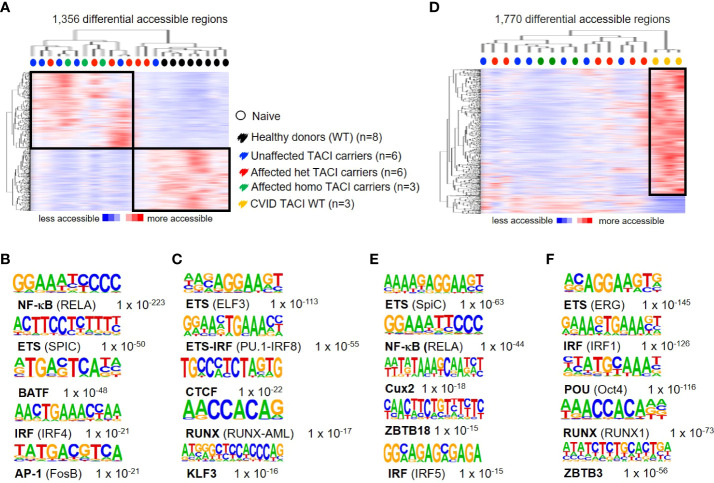
Dysregulated chromatin landscape in unstimulated naïve B cells from TACI mutation carriers. **(A)** Heatmap including hierarchical clustering of 1,356 differentially accessible chromatin regions in TACI mutation carriers compared to WT. Preferentially open regions amounted to 743 in TACI mutation carriers and 613 in WT. **(B, C)** Cis-regulatory sequences associated with regions of the **(B)** TACI mutation carriers or **(C)** WT. **(D)** Heatmap including hierarchical clustering of 1,770 differentially accessible chromatin regions in TACI mutation carriers compared to CVID TACI WT. Preferentially open regions amounted to 223 in TACI mutation carriers and 1,547 in CVID TACI WT. **(E, F)** Cis-regulatory sequences associated with the regions of the **(E)** TACI mutation carriers or **(F)** CVID TACI WT. Genes were normalized and centered with Cluster 3.0 and visualized with Java Treeview with color indicating the region with maximum (red) or minimum (blue) open chromatin. Sequence logos and *p*-values reflecting the significance of motif occurrence are shown next to the corresponding motif.

To address differences in chromatin accessibility in unstimulated naïve B cells from TACI mutation carriers, we generated the following TACI mutation carrier comparisons: heterozygous affected vs. homozygous affected and heterozygous affected vs. heterozygous unaffected mutation carriers. No DARs were detected in both comparisons with the previously described options. Therefore, all TACI mutation carriers were considered as one group for further investigations. We identified 1,356 DARs, with 743 regions more accessible in TACI mutation carriers (**Figure 2A**) and 613 DARs preferentially accessible in healthy donors. As observed in **Figure 1D**, TACI mutation carriers were distributed within a distinct cluster, facing a healthy donor cluster. TFBM analysis revealed NF-κB (**Figure 2B**) and ETS (**Figure 2C**) as the most enriched motifs in TACI mutation carriers and healthy donors, respectively. RELA is crucial for the generation of antigen-specific plasma cells in the GC reaction *in vivo* and SpiC opposes SpiB function, regulating B-cell function and differentiation (39, 40). Comparing CSM B cells from TACI mutation carriers to healthy donors revealed NF-κB as the most enriched TFBM as well (**Supplementary Figure 1**). To validate the previous findings and clarify whether this dysregulated enhancer repertoire was associated with the CVID phenotype in general, or the TACI mutational status in particular, naïve B cells from three CVID patients with wild-type TACI expression were included for comparison (**Figure 2D**). Surprisingly, we identified more DARs (1,770 regions) in this comparison than in the previous analysis with healthy donors. Eighty-five percent of DARs (1,547 regions) were accessible in CVID wild type for *TNFRSF13B*/TACI, while 223 DARs were accessible in TACI mutation carriers. The most enriched TFBM in TACI mutation carriers was SPIC followed by RelA (**Figure 2E**), which is consistent with the previous comparison between B-cell subpopulations in healthy donors. On the other hand, our results revealed TFBM for ERG and IRF1 as the most enriched in CVID patients without a mutation in *TNFRSF13B*/TACI (**Figure 2F**).

### Transcriptome landscape of unstimulated naïve B cells from TACI mutation carriers

**Figure 3:**
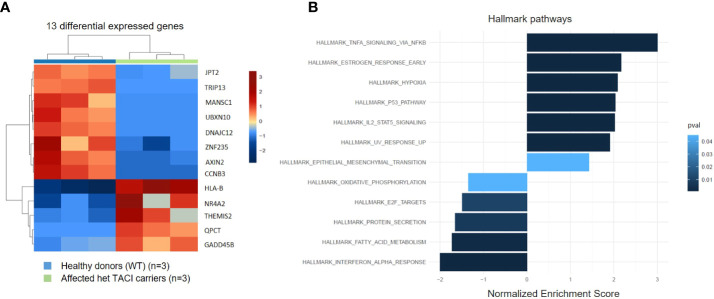
Expression pattern of differentially expressed genes from unstimulated naïve B cells from TACI mutation carriers and healthy donors. **(A)** Heatmap of DEG on RNA level for unstimulated naïve B cells from TACI mutation carriers and WT (log2 fold change >0.5) Red: upregulated genes; blue: downregulated genes. **(B)** Statistically over-represented Hallmark pathways associated with increased (right bars) or decreased (left bars) DEG depicted by the histogram.

To identify alterations in the transcriptome of CVID patients harboring the C104R TACI mutation, we performed RNA-seq and analyzed unstimulated naïve B cells from three affected TACI mutation carriers and three healthy donors. The differentially expressed genes (DEGs) were determined with DESeq2. The transcripts were filtered according to their adjusted *p*-value (<0.05) and log2 fold change (>0.5) revealing five genes (*HLA-B*, *NR4A2*, *THEMIS2*, *QPCT*, and *GADD45B*) significantly upregulated and eight genes (*CCNB3*, *AXIN2*, *ZNF235*, *DNAJC12*, *UBXN10*, *MANSC1*, *TRIP13*, and *JPT2*) significantly downregulated (**Figure 3A**). In CSM B cells, we found fewer DEGs (**Supplementary Figure 2B**). To estimate the influence of the DEGs, we performed gene ontology enrichment analysis including DEGs with *p*-value > 0.05, considering Hallmark pathways (**Figure 3B**). The TNF-α signaling *via* the NF-κB pathway presented with the highest enrichment, followed by pathways involved in cell death (hypoxia, p53 signaling, and UV response). NF-κB is a key player in peripheral immune tolerance in secondary hematopoietic organs, circulating blood, and control genes involved in anti-apoptotic mechanisms (41). p53 serves as a sensor of cellular stress, responding to irradiation-induced DNA damage, hypoxia, oncogene expression, nutrient deprivation, and ribosome dysfunction, and limiting the expansion of cells under these conditions (42). The most downregulated pathways were interferon-α response, followed by pathways influencing cell metabolism (fatty acid metabolism, protein secretion, and oxidative phosphorylation). Recent studies have determined alterations in these pathways in CVID patients (43–45). In CSM B cells, we found the same dysregulated pathways and more (**Supplementary Figure 2B**).

### Influence of CD40L and IL21 stimulation on the transcriptome of naïve B cells from TACI mutation carriers

**Figure 4:**
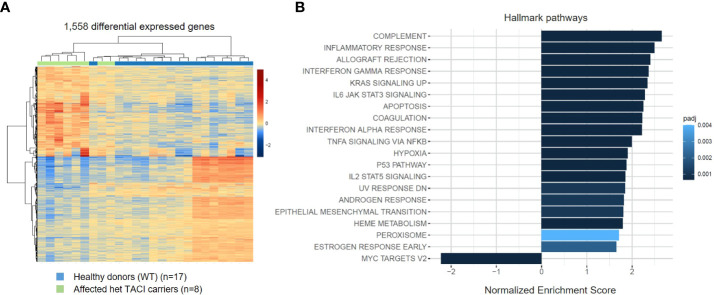
Transcriptome analysis of stimulated naïve B cells from TACI mutation carriers and healthy donors. **(A)** Heatmap of DEG on RNA level for stimulated naïve B cells from TACI mutation carriers and WT (log2 fold change >0.5) Red: upregulated genes; blue: downregulated genes. **(B)** Statistically over-represented Hallmark pathways associated with increased (right bars) or decreased (left bars) DEG depicted by the histogram.

To investigate the influence of impaired NF-κB signaling upon activation, we generated transcriptomes of CD40L and IL21 stimulated naïve B cells from eight affected TACI mutation carriers and 17 healthy donors. Next, we analyzed the datasets with the same conditions as for unstimulated naïve B cells. In contrast to the unstimulated analysis, 1,558 DEGs were detected in the TACI mutation carriers (**Figure 4A**), 720 upregulated and 838 downregulated. Then, we performed gene ontology enrichment analysis, considering Hallmark pathways (**Figure 4B**). In contrast to unstimulated naïve B cells from affected TACI mutation carriers, pathways involved in the immune response were the most enriched (complement, inflammatory response, and allograft rejection) after stimulation. All upregulated pathways detected in unstimulated B cells were found in stimulated B cells but with less impact (TNF-α signaling *via* NF-κB, hypoxia, p53 signaling, and UV response). The only downregulated pathway detected was on MYC-Version 2 (V2) target genes. MYC is a TF that regulates multiple human genes that promote cell growth and proliferation (46). It can alter apoptosis and activation of telomerase, and it controls angiogenesis (47).

These findings suggest that dysregulated mechanisms found in steady-state naïve B cells persist throughout maturation and is accompanied by over-activation of immune responses.

### Proteome analysis of stimulated naïve B cells from TACI mutation carriers

**Figure 5:**
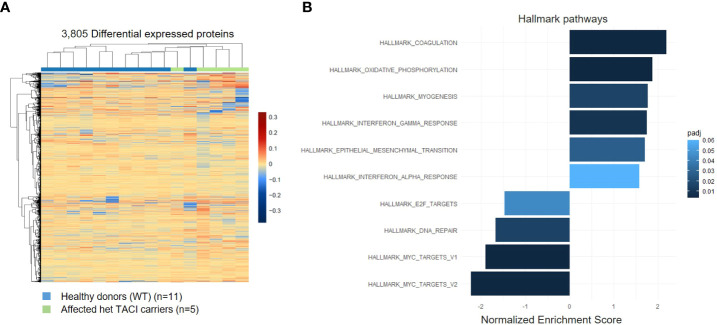
Proteome analysis of stimulated naïve B cells from TACI mutation carriers and healthy donors. **(A)** Heatmap of differentially expressed proteins for stimulated naïve B cells from TACI mutation carriers and WT (log2 fold change >0.5) Red: upregulated proteins; blue: downregulated proteins. **(B)** Statistically over-represented Hallmark pathways associated with increased (right bars) or decreased (left bars) differentially expressed proteins depicted by the histogram.

As the presence of RNA transcripts does not necessarily lead to protein abundance, we examined the proteome of CD40L and IL21 stimulated naïve B cells from five affected TACI mutation carriers and 11 healthy donors. All individuals were included in the transcriptome analysis. With the specification >0.5 log2 fold change and adjusted *p*-value <0.05, 3,805 differentially expressed proteins were detected in the TACI mutation carriers, 1,768 upregulated and 2,037 downregulated (**Figure 5A**). Gene ontology enrichment analysis identified dysregulated pathways using Hallmark genes. The majority of upregulated pathways overlapped with the transcriptome analysis, such as coagulation, interferon-α (IFN-α), and interferon-γ (IFN-γ) (**Figure 5B**). Coagulation is supported by platelets (48), which are capable of delivering CD154 to sites distant from the location of activation to stimulate antigen-specific IgG production (49). IFN-α and -γ enable B cells to undergo GC formation and isotype-switching, and mature into ASC (50, 51). In contrast to unstimulated naïve B cells, we found the pathway “oxidative phosphorylation” enriched. On the other hand, the MYC-V2 target gene pathway was decreased, as detected in the transcriptome of stimulated naïve B cells. Furthermore, we observed a reduction of the E2F targets, which were found downregulated at the transcriptome level at resting state and the DNA repair pathways. E2F family members play a major role during the G1/S transition in mammals, and E2F directly links cell cycle progression with the coordinate regulation of genes essential for both the synthesis of DNA and its surveillance (52).

### Chromatin accessibility maps of stimulated naïve B cells from TACI mutation carriers

**Figure 6:**
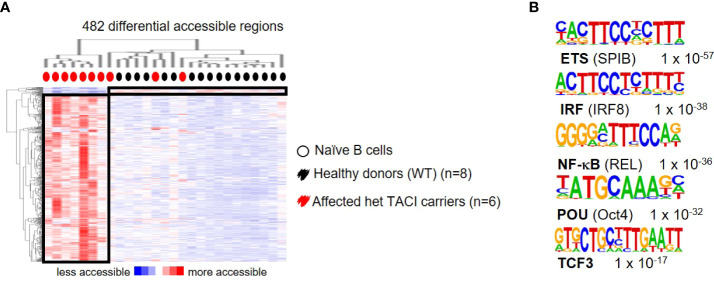
Dysregulated chromatin landscape in stimulated naïve B cells from TACI mutation carriers. **(A)** Heatmap including hierarchical clustering of 482 differentially accessible chromatin regions in TACI mutation carriers compared to WT. Preferentially open regions amounted to 465 in TACI mutation carriers and 17 in WT. **(B)** Cis-regulatory sequences associated with regions of the TACI mutation carriers. Transcription factor family stated first and in brackets the family member. Genes were normalized and centered with Cluster 3.0 and visualized with Java Treeview with color indicating the region with maximum (red) or minimum (blue) open chromatin. Sequence logos and *p*-values reflecting the significance of motif occurrence are shown next to the corresponding motif.

To further explore the dysregulation in affected TACI mutation carriers, we stimulated naïve B cells from 10 patients and 17 healthy donors with CD40L and IL21 for 24 h and analyzed their chromatin landscape. A total of 482 differentially accessible regions (DAR) were identified (using 10-fold greater tag density than background), with the majority (465) accessible in affected TACI mutation carriers (**Figure 6A**), while 17 DARs were detected in healthy donors. To identify TFs mediating this differential chromatin accessibility, we performed *de novo* motif analysis using HOMER. The most enriched TFBM with lengths 8, 10, 12, and 15 bp and a peak size 200 were SPI-B, IRF8, REL, Oct4, and TCF3 (**Figure 6B**). All detected TFs with enriched binding motifs are important regulators of B cell differentiation and proliferation. REL (cREL) is required for GC formation, maintenance, and cell proliferation (53). TCF3 acts primarily as a transcriptional activator in B cells, and controls as well as aids the expression of genes critical for GC and B cell development (54). Monogenic mutations in TCF3 are known to cause antibody deficiency by homozygous and heterozygous mutations (55–58). Moreover, TCF3 has been observed to produce an epistatic effect when mutated together with TACI/TNFRSF13B (59). No TFBM enrichment was detectable in the healthy donors, due to the low amount of DARs.

### Functional validation of NF-κB and pERK dysregulation

**Figure 7:**
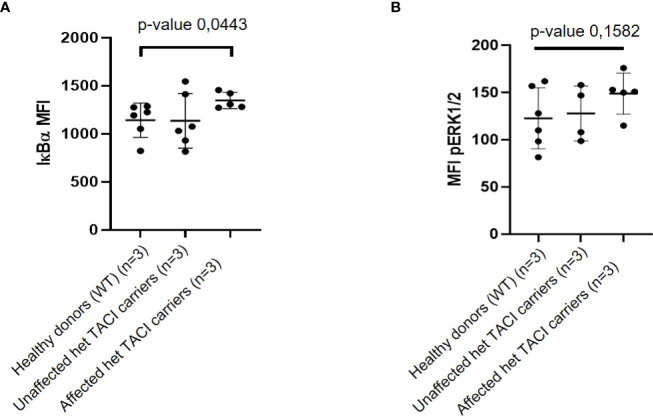
IκBα and pERK expression in unstimulated naïve B cells from TACI mutation carriers. **(A)** Mean fluorescence intensity (MFI) of IκBα in naïve B cells from affected and unaffected TACI mutation carriers and healthy donor. **(B)** Mean fluorescence intensity (MFI) of pERK in naïve B cells from affected and unaffected TACI mutation carriers and healthy donor. *p*-values (*p < 0.05) derived from unpaired *t*-test calculated with GraphPad Prism 8.

To investigate whether the enriched NF-κB motifs in unstimulated naïve B cells from TACI mutation carriers suggested *via* ATAC-seq result from NF-κB signaling alterations, we determined IκBα degradation as described by Keller et al. (24). To this end, we analyzed PBMCs from three affected and three unaffected TACI mutation carriers and three healthy donors. Two samples from different time-points from each individual were obtained for analysis. Using the Grubbs’ test, one outlier (*Z*-score = 1.98) was identified among naïve B cells from affected TACI mutation carriers and excluded from the analysis. Our analysis revealed an elevation of basal IκBα levels in naïve B cells from affected TACI mutation carriers (**Figure 7A**). However, IκBα expression in naïve B cells from affected and unaffected TACI mutation carriers stimulated for short periods of time (15–30 min) with different NF-κB signaling initiators (α-IgM, CpG, PMA, and CD40L) was similar to healthy donors (**Supplementary Figures 3A, B**).

By integrating the upregulated DEGs (312, *p*-value < 0.05) from unstimulated naïve B cells and chromatin regions harboring the enriched TFBM for NF-κB (50), four genes were identified in both datasets: MAP3K8, COTL, PPIF, and G3PB2. MAP3K8 is an enzyme that activates both ERK1/2 and p38 MAP kinases, which are involved in the regulation of meiosis, mitosis, and postmitotic functions, and response to stress stimuli, respectively (60). To functionally validate the observed dysregulation of MAP3K8, we investigated the phosphorylation state of ERK. ERK is phosphorylated downstream of MAP3K8 activation and transduces CD40 signaling ***via*** the NF-κB pathway (61). Thus, we analyzed PBMCs from three affected and three unaffected TACI mutation carriers and three healthy donors (same individuals as for the IκBα assay). Two samples from different time points from each individual were obtained for this analysis. Due to limited cell numbers in the flow cytometry analysis, one naïve B-cell sample from an affected mutation carrier and two naïve B-cell samples from an unaffected carrier were excluded from the analysis. In naïve B cells, the basal expression of pERK was slightly increased in affected TACI mutation carriers compared to healthy donors, while the unaffected TACI mutation carriers showed intermediate pERK expression (**Figure 7B**). After stimulation with CD40L for 8 min, pERK expression showed an increase in affected TACI mutation carriers, while unaffected carriers expressed pERK similar to healthy donors (**Supplementary Figure 4**). These results support the dysregulation of NF-κB and ERK signaling in naïve B cells of C104R TACI mutation carriers and may possibly point towards novel targets not only for the treatment of TACI mutation carriers but also for patients with CVID of unknown genetic cause and patients with lymphoproliferation and autoimmunity in general, which is the predominant phenotype seen in our C104R-TACI-mutated individuals.

## Discussion

Over the last decades, multiple attempts have been made to unravel the puzzling immunodeficiency termed “CVID”. With the rise of the NGS technology, such as whole genome, whole exome, or targeted gene panel sequencing, several monogenetic mutations have been identified, causative for the CVID phenotype. However, only approximately 20%–30% of CVID patients harbor monogenetic mutations explaining their clinical features, indicating that there are additional mechanisms involved in disease development. Therefore, polygenic alterations, modifier genes, and/or epigenetic and/or environmental factors could play leading roles in CVID development and/or progression. In our study, we combined chromatin accessibility mapping, transcriptome, and proteome analysis in unstimulated and stimulated B naïve B cells of CVID patients harboring the C104R mutation in TACI to gain insights into their immune dysregulation syndrome. We focused on C104R mutation carriers, as this mutation is by far the most frequent genetic mutation detected in CVID patients (62), albeit with a knowingly reduced penetrance. We hypothesized that by comparing affected and unaffected C104R mutation carriers, we would detect a difference by comparing their epigenetic, transcriptomic, and proteomic signatures to potentially put us into the position to be able to predict which C104R mutation carrier has to expect disease manifestation.

We identified dysregulated chromatin landscapes in unstimulated and stimulated naïve B cells involving binding sites for TFs known for their importance in the GC reaction, such as NF-κB, ETS, and IRF family members. Under both conditions, factors with negative roles for B-cell development and function (SPI-C in unstimulated and IRF8 in stimulated naïve B cells) showed enriched TFBM, which might contribute to impaired memory B-cell development (63, 64). The observed chromatin landscape seems TACI mutation-associated rather than CVID-specific, as the chromatin accessibility of three CVID patients with WT-TACI expression differed from TACI mutation carriers. Future experiments investigating the transcriptome and proteome comparing TACI mutation carriers, CVID patients with WT-TACI, and CVIDs with monogenic disease-causing mutations (such as NFKB1 or CTLA4) may shed light on the specific dysregulations associated with their genetic cause.

In addition, our transcriptome results from naïve B cells highlighted an increase of different pathways able to induce programmed cell death. An increase of radiosensitivity in CVID patients, leading to the elevation of chromosomal aberrations, has been reported (65). In contrast to our study, stimulation of total CD19+ B cells from CVID patients with CpG revealed p53 induction, preventing spontaneous and irradiation-induced cell death (66). Our data indicate improved cell survival in unstimulated naïve B cells in TACI mutation carriers, supporting the development of autoimmunity. These transcriptome alterations proceeded after CD40L and IL-21 stimulation.

Impaired metabolic processes in steady-state naïve B cells and reduced cell survival signaling in activated naïve B cells are in agreement with the observation that in CVID patients, B cells fail to perform in the GC reaction and hence class-switched memory B cells are missing in CVID patients (1). Furthermore, proteome analysis supported the lack of proper cell activation and cell survival, as MYC and E2F target gene pathways were decreased. Our findings verify observations reported decades ago, describing absent or reduced expression of MYC in B cells from CVID patients (67). A recent report showed a reduction of E2F signaling in naïve and CD21low B cells from CVID patients, further pointing towards a CVID-associated alteration (45) and possibly accounting for the reduced B-cell survival in patients. The increased interferon-α and -γ response in stimulated naïve B cells from TACI mutation carriers provides another cause for autoimmune manifestation: It was shown that the enhanced interferon signature in naïve B cells from SLE patients drives autoimmunity (51, 68). In addition, CD21low B cells represent a B-cell subpopulation with an elevated interferon response (69), highlighting the similarities between CD21low B cells and naïve B cells carrying the TACI mutation. We showed dysregulation of NF-κB signaling at the chromatin and transcriptional level, further supported by increased IκBα and pERK expression, observations made in multiple other published studies and known to facilitate CVID development (24, 70, 71). We hypothesize that naïve B cells from TACI CVID patients are in a transcriptionally and epigenetically activated state, leading to an increase in NF-κB signaling, which, in turn, results in an impaired B-cell development. Through CD40L and IL-21 stimulation, these naïve B cells mount an overactivation as demonstrated by the enhanced inflammatory- and interferon response, which suppresses MYC signaling and hinders cell proliferation.

Nevertheless, future studies will need to confirm our observations, as the cohort of investigated patients was small. Furthermore, the diverse genetic and epigenetic landscapes of B cells from the single C104R carriers observed in our multi-omics approach bias the detection of differentially expressed transcripts, proteins, and chromatin accessibility. A precise selection of TACI mutation carriers used for investigation could identify additional alterations. With the use of single-cell sequencing, individual B-cell profiles from CVID patients with unknown genetic cause started to unravel novel insights into functional alterations, usually masked by bulk sampling methods (72). This technology (scRNA-Seq) would benefit further investigations in TACI mutation carriers.

Together, our study described a multi-omics approach to investigate altered B-cell development in CVID patients harboring a TACI mutation combining chromatin accessibility, transcriptome, and proteome analysis. The observed increase of apoptotic pathways and NF-κB signaling impairment will account for the lack of appropriate memory B-cell development. Furthermore, the reduction of cell cycle gatekeepers MYC and E2F target genes contributes to the impaired proliferation and survival of naïve B cells. These findings not only provide insights into the pathophysiology of CVID, but also reveal novel pathways for further investigations, and may hence help to develop targeted therapy strategies for patients with mutations in *TNFRSF13B*.

## Data availability statement

The NGS datasets presented in this article (RNA-seq and ATAC-seq) have been deposited on the SRA database (BioProject ID: PRJNA859010). The mass spectrometry proteomics data have been deposited to the ProteomeXchange Consortium via the PRIDE (73) partner repository with the dataset identifier PXD035459.

## Ethics statement

The studies involving human participants were reviewed and approved by Ethics committee of the University of Freiburg, Germany. The patients/participants provided their written informed consent to participate in this study.

## Author contributions

NR processed and collected samples, performed ATAC-seq, RNA-seq proteome analysis, functional assays, designed layout, figures and tables, and wrote the manuscript. SP-C processed and collected samples, and designed RNA-seq script. NL implemented ATAC-seq protocol and collected samples. AC analyzed and interpreted WES data for TACI mutation confirmation. MPr analyzed and interpreted WES, designed sorting strategy, and revised the work critically for intellectual content. BK implemented functional assays and interpretation. FZ sequenced ATAC-seq samples and data analysis. VG processed ATAC-seq samples. MPe measured proteome samples and revised analysis. HE conceived and designed the study and revised the work critically for intellectual content. KW cared for patients, provided immunological information, and revised the work critically for intellectual content. EB conceived and designed the study, and revised the work critically for intellectual content. RG conceived and designed the study, and performed measurement of proteome. CB conceived and designed the study and performed ATAC-seq implementation and analysis. BG conceived and designed the study, provided and cared for study patients, and revised the work critically for intellectual content. All authors contributed to manuscript revision, read, and approved the submitted version.

## Funding

BG receives support from the Deutsche Forschungsgemeinschaft (DFG) SFB1160/2_B5, under Germany’s Excellence Strategy (CIBSS – EXC-2189 – Project ID 390939984, and RESIST – EXC 2155 – Project ID 390874280); the E-rare program of the EU, managed by the DFG, grant code GR1617/14-1/iPAD; and the German Federal Ministry of Education and Research (BMBF) through a grant to the German Auto-Immunity Network (GAIN), grant code 01GM1910A. This work was supported in part by the Center for Chronic Immunodeficiency (CCI), Freiburg Center for Rare Diseases (FZSE). Some samples have been taken from the CCI-biobank, a partner of the Freeze Biobank Freiburg. The article processing charge was funded by the Baden-Wuerttemberg Ministry of Science, Research and Art and the University of Freiburg in the funding programme Open Access Publishing.

## Acknowledgments

We thank all affected individuals, their unaffected family members, and healthy volunteers who participated in this study. We thank Pavla Mrovecova, Katrin Hübscher, and Hanna Haberstroh for excellent technical assistance. We are grateful to Pascal Schneider for providing purified CD40L. We acknowledge the Lighthouse Core Facility for their excellent assistance with cell sorting and flow cytometry. Some samples have been taken from the CCI-biobank, a partner of the Freeze Biobank Freiburg. We thank Christoph Bock and the Biomedical Sequencing Facility at CeMM for assistance with next-generation sequencing, as well as Novogene and EMBL. The authors acknowledge the support of the Freiburg Galaxy Team: Beatriz Serrano-Solano and Björn Grüning, Bioinformatics, University of Freiburg (Germany) funded by the Collaborative Research Centre 992 Medical Epigenetics (DFG grant SFB 992/1 2012) and the German Federal Ministry of Education and Research BMBF grant 031 A538A de.NBI-RBC. We appreciate Biorender with whom we have drawn the graphical abstract.

## Conflict of interest

BG is employed by the University Hospital Freiburg, Germany. He currently receives funding for his research from the following third parties: Deutsche Forschungsgemeinschaft (DFG); the E-rare program of the EU, managed by the DFG; the “Netzwerk Seltener Erkrankungen” of the German Ministry of Education and Research (BMBF); Merck KGaA; Takeda Pharma Vertrieb GmbH & Co. KG; Bristol-Myers Squibb GmbH & Co. KGaA; Novartis Pharma AG; and CSL Behring GmbH. This work was supported in part by the Center for Chronic Immunodeficiency (CCI), Freiburg Center for Rare Diseases (FZSE). During the last 2 years, BG was an advisor to the following companies: UCB Pharma S.A., Epimune GmbH, Octapharma, Atheneum Partners GmbH, and GigaGen Inc. and a speaker for Janssen-Cilag GmbH (receiving fees less than 1,000€).

The remaining authors declare that the research was conducted in the absence of any commercial or financial relationships that could be construed as a potential conflict of interest.

## Publisher’s note

All claims expressed in this article are solely those of the authors and do not necessarily represent those of their affiliated organizations, or those of the publisher, the editors and the reviewers. Any product that may be evaluated in this article, or claim that may be made by its manufacturer, is not guaranteed or endorsed by the publisher.

## Supplementary material

Supplementary Figure 1 Dysregulated chromatin landscape in CSM B cells from TACI mutation carriers. **(A)** Heatmap including hierarchical clustering of 1,069 differentially accessible chromatin regions in TACI mutation carriers compared to WT. Preferentially open regions amounted to 371 in TACI mutation carriers and 698 in WT. **(B, C)** Cis-regulatory sequences associated with regions of the **(B)** TACI mutation carriers or **(C)** WT.

Supplementary Figure 2 Expression pattern of differentially expressed genes from CSM B cells from TACI mutation carriers and healthy donors. **(A)** Heatmap of DEG on RNA-level for CSM B cells from TACI mutation carriers and WT (log2 fold change >0.5) Red: upregulated genes; blue: downregulated genes. **(B)** Statistically over-represented Hallmark pathways associated with increased (right bars) or decreased (left bars) DEG depicted by the histogram.

Supplementary Figure 3 IκBα expression in unstimulated and stimulated naïve B cells from TACI mutation carriers. **(A)** Mean fluorescence intensity (MFI) of IκBα in unstimulated and with CpG, anti-IgM, PMA and CD40L stimulated naïve B cells from affected and unaffected TACI mutation carriers and healthy donor. **(B)** Ratios of the MFI of IκBα after and without indicated stimulation. P-values (* P <0.05) derive from unpaired t-test calculated with GraphPad Prism 8. # n.s.: non-significant.

Supplementary Figure 4 pERK expression in unstimulated and stimulated naïve B cells from TACI mutation carriers. **(A)** Mean fluorescence intensity (MFI) of pERK in unstimulated and with CD40L stimulated naïve B cells from affected and unaffected TACI mutation carriers and healthy donor. **(B)** Ratios of the MFI of IκBα after and without indicated stimulation. P-values (* P <0.05) derive from unpaired t-test calculated with GraphPad Prism 8. # n.s.: non-significant.

## Abbreviations

ATAC-seq, Assay for Transposase-Accessible Chromatin using sequencing; CSM, Class switch memory; CVID, Common variable immunodeficiency; DEG, Differentially expressed genes; ETS, Erythroblast transformation specific; FACS, Fluorescence-activated cell sorting; GC, Germinal center; IRF, Interferon regulatory factor; NFκB, Nuclear factor kappa B subunit; PBMCs, Peripheral blood mononuclear cells; TACI, Transmembrane activator and calcium modulator and cyclophilin ligand; TF, Transcription factor; TFBM, Transcription factor binding motif; TNFRSF13B, Tumor necrosis factor receptor super family 13 B; WT, Wild type.
