# Supplementary figures and images for: Multi-omics analysis of naïve B cells of patients harboring the C104R mutation in TACI
Source: Front Immunol. 2022 Aug 16;13:938240. doi: 10.3389/fimmu.2022.938240 (PMC9443529; doi:10.3389/fimmu.2022.938240)

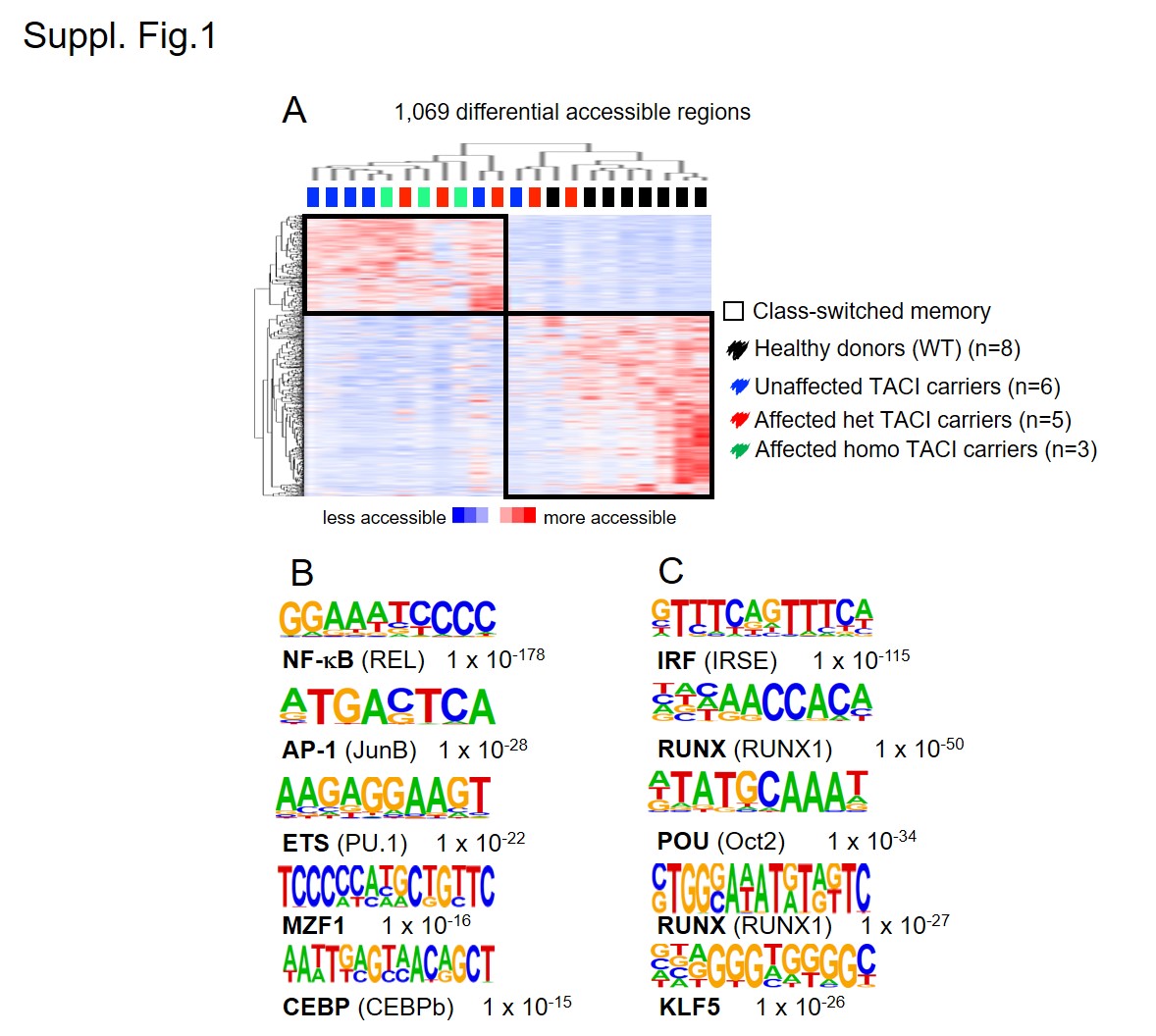

Supplement: Supplementary Figure 1 — Dysregulated chromatin landscape in CSM B cells from TACI mutation carriers. (A) Heatmap including hierarchical clustering of 1,069 differentially accessible chromatin regions in TACI mutation carriers compared to WT. Preferentially open regions amounted to 371 in TACI mutation carriers and 698 in WT. (B, C) Cis-regulatory sequences associated with regions of the (B) TACI mutation carriers or (C) WT. [file Image_1.jpg]

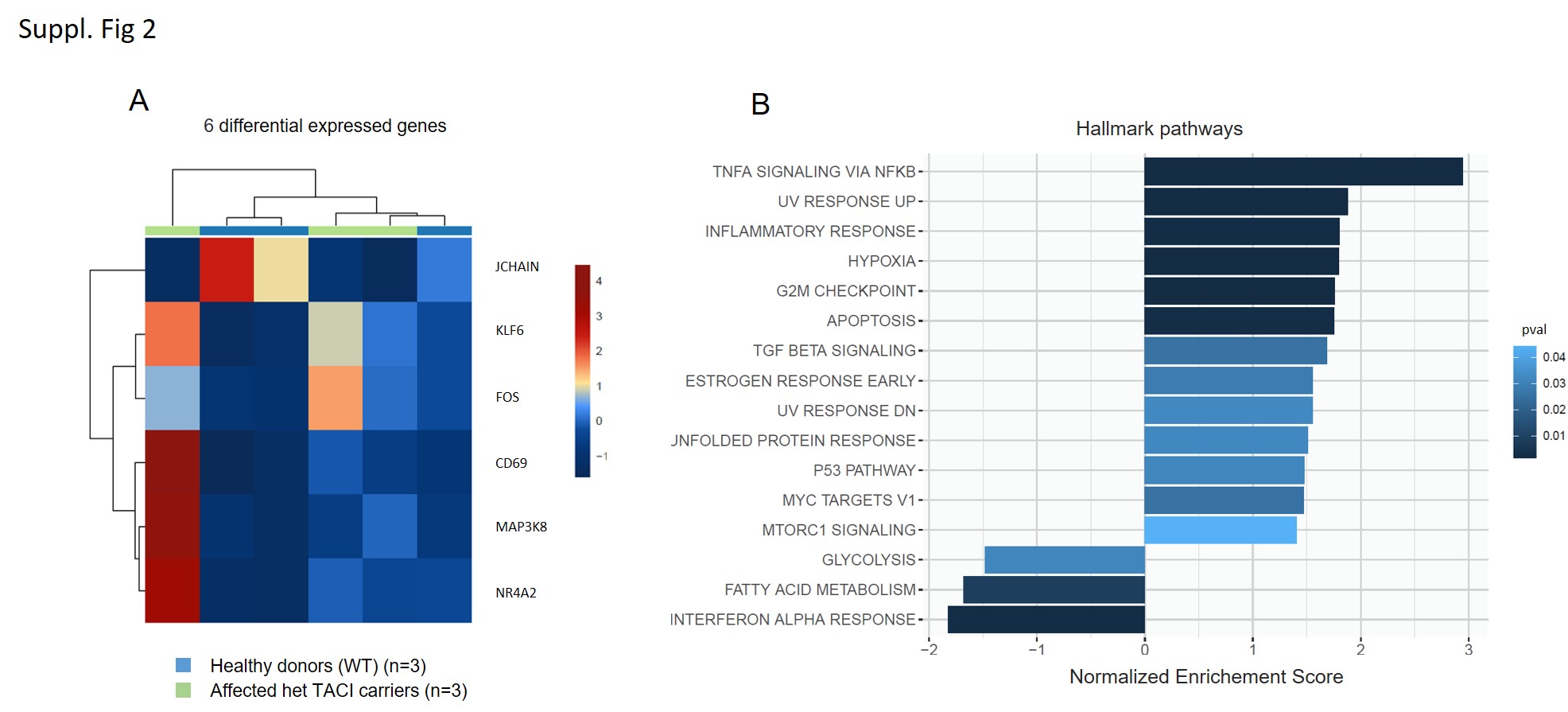

Supplement: Supplementary Figure 2 — Expression pattern of differentially expressed genes from CSM B cells from TACI mutation carriers and healthy donors. (A) Heatmap of DEG on RNA-level for CSM B cells from TACI mutation carriers and WT (log2 fold change >0.5) Red: upregulated genes; blue: downregulated genes. (B) Statistically over-represented Hallmark pathways associated with increased (right bars) or decreased (left bars) DEG depicted by the histogram. [file Image_2.jpg]

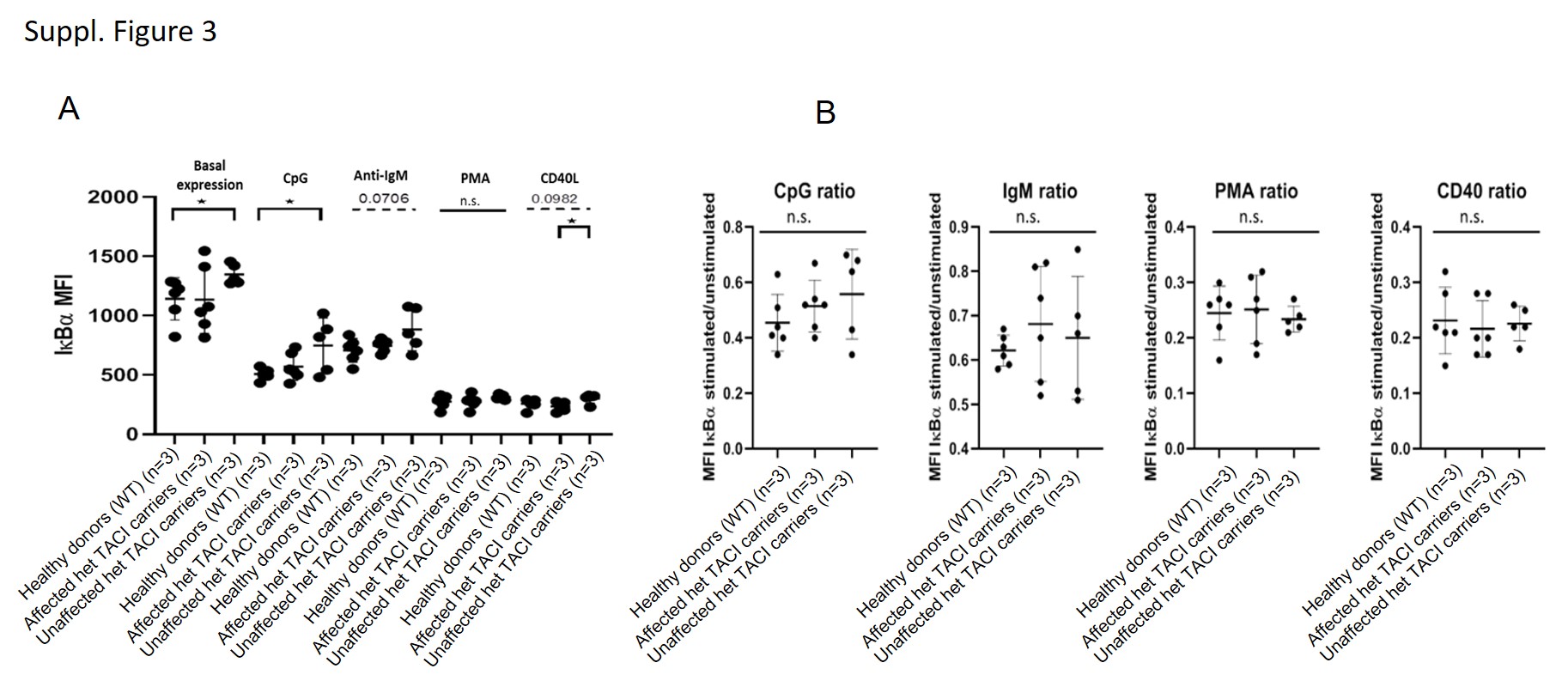

Supplement: Supplementary Figure 3 — IκBα expression in unstimulated and stimulated naïve B cells from TACI mutation carriers. (A) Mean fluorescence intensity (MFI) of IκBα in unstimulated and with CpG, anti-IgM, PMA and CD40L stimulated naïve B cells from affected and unaffected TACI mutation carriers and healthy donor. (B) Ratios of the MFI of IκBα after and without indicated stimulation. P-values (* P <0.05) derive from unpaired t-test calculated with GraphPad Prism 8. # n.s.: non-significant. [file Image_3.jpg]

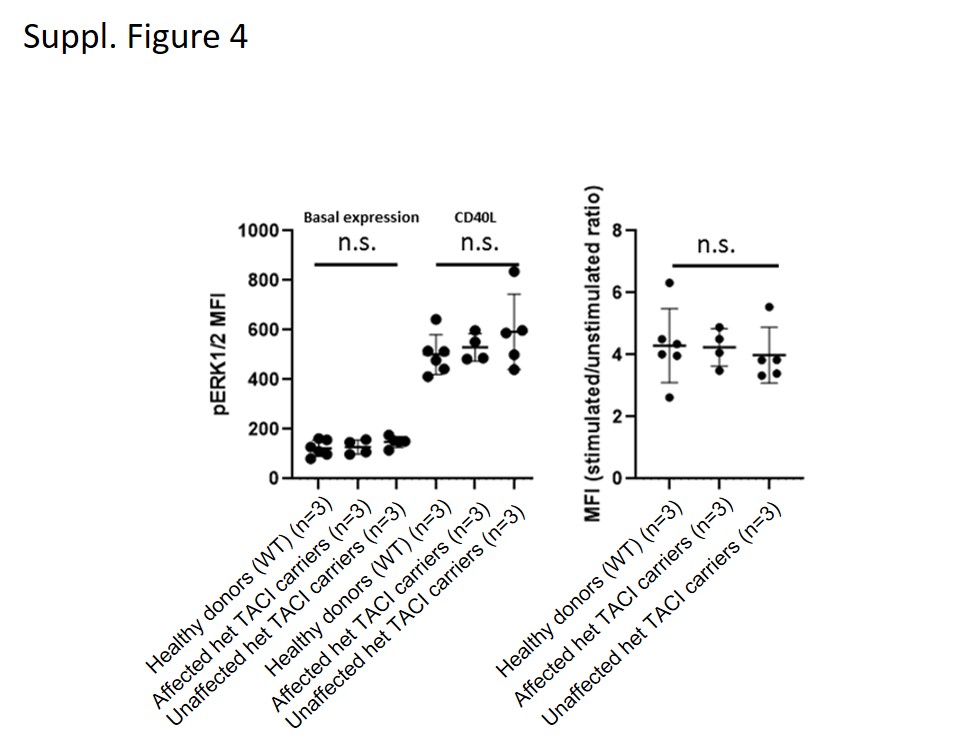

Supplement: Supplementary Figure 4 — pERK expression in unstimulated and stimulated naïve B cells from TACI mutation carriers. (A) Mean fluorescence intensity (MFI) of pERK in unstimulated and with CD40L stimulated naïve B cells from affected and unaffected TACI mutation carriers and healthy donor. (B) Ratios of the MFI of IκBα after and without indicated stimulation. P-values (* P <0.05) derive from unpaired t-test calculated with GraphPad Prism 8. # n.s.: non-significant. [file Image_4.jpg]

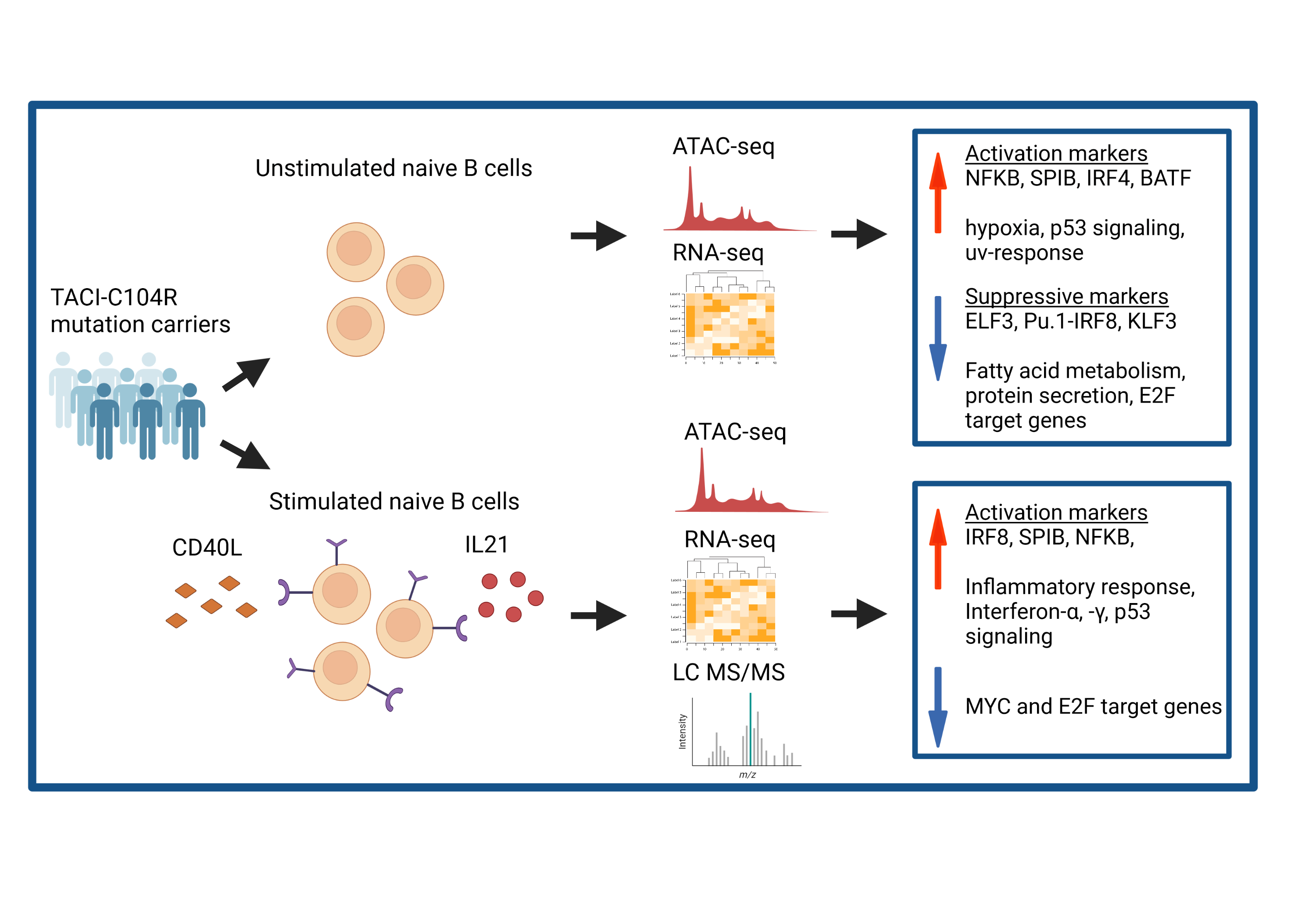

Supplement: Supplementary file 5 [file Image_5.png]
